# Effect of aerobic training on baseline expression of signaling and respiratory proteins in human skeletal muscle

**DOI:** 10.14814/phy2.13868

**Published:** 2018-09-10

**Authors:** Daniil V. Popov, Evgeny A. Lysenko, Roman O. Bokov, Maria A. Volodina, Nadia S. Kurochkina, Pavel A. Makhnovskii, Mikhail Y. Vyssokikh, Olga L. Vinogradova

**Affiliations:** ^1^ Laboratory of Exercise Physiology Institute of Biomedical Problems of the Russian Academy of Sciences Moscow Russia; ^2^ Faculty of Fundamental Medicine M.V. Lomonosov Moscow State University Moscow Russia; ^3^ Laboratory of Mitochondrial Medicine Research Center for Obstetrics Gynecology and Perinatology Ministry of Healthcare of the Russian Federation Moscow Russia

**Keywords:** aerobic training, baseline expression, exercise, gene expression, human skeletal muscle, mitochondrial respiratory proteins, transcription regulators

## Abstract

Most studies examining the molecular mechanisms underlying adaptation of human skeletal muscles to aerobic exercise focused on the response to acute exercise. Here, we examined the effect of a 2‐month aerobic training program on baseline parameters in human muscle. Ten untrained males performed a one‐legged knee extension exercise for 1 h with the same relative intensity before and after a 2‐month aerobic training program. Biopsy samples were taken from vastus lateralis muscle at rest before and after the 2 month training program (baseline samples). Additionally, biopsy samples were taken from the exercised leg 1 and 4 h after the one‐legged continuous knee extension exercise. Aerobic training decreases baseline phosphorylation of FOXO1^Ser256^, increases that of CaMKII^T^ ^hr286^, CREB1^Ser133^, increases baseline expression of mitochondrial proteins in respiratory complexes I–V, and some regulators of mitochondrial biogenesis (TFAM, NR4A3, and CRTC2). An increase in the baseline content of these proteins was not associated with a change in baseline expression of their genes. The increase in the baseline content of regulators of mitochondrial biogenesis (TFAM and NR4A3) was associated with a transient increase in transcription after acute exercise. Contrariwise, the increase in the baseline content of respiratory proteins does not seem to be regulated at the transcriptional level; rather, it is associated with other mechanisms. Adaptation of human skeletal muscle to regular aerobic exercise is associated not only with transient molecular responses to exercise, but also with changes in baseline phosphorylation and expression of regulatory proteins.

## Introduction

Regular aerobic physical exercise (aerobic training) increases capillary density, mitochondrial volume density, and oxidative capacity in skeletal muscles. These adaptive changes lead to a reduction in the rate at which products of glycolysis accumulate in working muscle and increase muscle maximal oxygen delivery and uptake, thereby increasing the aerobic performance of muscles and of the organism as a whole. The molecular mechanisms underlying adaptation of skeletal muscle to aerobic exercise have been studied extensively. Acute exercise induces transient changes in phosphorylation of kinases (Hoffman et al. 2015); the most important kinases that facilitate adaptation to aerobic exercise are AMP‐activated protein kinase (AMPK), Ca^2+^/calmodulin‐dependent protein kinases (CaMKs), and mitogen‐activated protein kinases p38 MAPK and ERK1/2. Acute exercise‐induced activation of signaling pathways alters the activity of coactivators and transcription factors, along with expression of genes that regulate angiogenesis, mitochondrial biogenesis, carbohydrate and lipid metabolism, and proteolysis. Many studies show the important role of PGC‐1*α* coactivator (encoded by the *PPARGC1A* gene) and mitochondrial transcription factor A (TFAM) in regulating mitochondrial biogenesis and adaptation of rodent and human skeletal muscles to aerobic exercise (Scarpulla 2008; Olesen et al. 2010). Experiments designed to examine changes in gene expression by murine myoblasts identified other regulators responsible for activating mitochondrial biogenesis. These include nuclear receptor subfamily 4 group A member 3 (NR4A3) (Pearen and Muscat 2018), nuclear receptor corepressor 1 (NCOR1) (Yamamoto et al. 2011; Pérez‐Schindler et al. 2012), CREB‐regulated transcription coactivators (CRTCs) (Wu et al. 2006), and estrogen‐related receptor gamma (ESRRG) (Rangwala et al. 2010; Narkar et al. 2011). However, the role of these proteins in adaptation of human skeletal muscle to aerobic exercise remains poorly understood. Studies investigating the transcriptome in human skeletal muscle report very pronounced (Mahoney et al. 2005; Catoire et al. 2012; Neubauer et al. 2013; Popov et al. 2015) and intensity‐dependent (Popov et al. 2018) increases in expression of the *NR4A3*,* ESRRG,* and *PPARGC1A* genes after acute exercise. These observations suggest that regular aerobic exercise increases the basal amounts of these proteins in human muscle.

Most studies examining the molecular mechanisms underlying adaptation of human skeletal muscles to aerobic exercise focused on the response to acute exercise; thus less focus has been placed on training‐induced changes. Changes in the phosphorylation and activity of regulatory proteins, as well as in expression of different genes in response to acute exercise, are transient. Therefore, it can be assumed that a small but permanent increase in the baseline level of phosphorylation activity, along with the amounts of regulatory proteins, plays an equally important role in adaptation of skeletal muscle to regular exercise.

Here, we examined the effect of a 2‐month aerobic training program on baseline parameters in human skeletal muscle, namely, phosphorylation of kinases ACC1/2^Ser79/222^ (a marker of AMPK activity), CaMKII^Thr286^, p38 MAPK^Thr180/Tyr182^ and ERK1/2^Thr202/Tyr204^, phosphorylation of CREB1^Ser133^ and FOXO1^Ser256^ and the amounts of transcriptional regulators PGC‐1*α*, TFAM, ESRRG, NR4A3, CRTC2, and NCOR1, and mitochondrial proteins in respiratory complexes I–V. To identify the mechanisms underlying changes in the cellular content of these proteins, we examined baseline expression of their encoding genes and changes in responses to acute aerobic exercise before and after 2 months of aerobic exercise.

## Materials and Methods

### Study design

**Figure 1:**
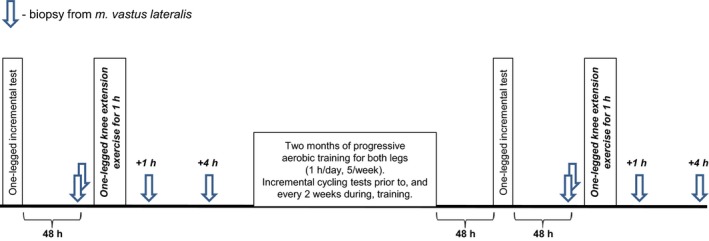
Study design. Ten untrained males performed a one‐legged continuous knee extension exercise with the same relative intensity before and after a 2‐month aerobic training program. The intensity of the one‐legged continuous knee extension exercise was chosen using an incremental one‐legged ramp test. Biopsy samples were taken from either leg (biological replicates) at rest before and after the 2‐month training program (baseline samples). Additionally, biopsy samples were taken from the exercised leg 1 and 4 h after the one‐legged continuous knee extension exercise.

The study was approved by the Ethics Committee of the Institute and complied with the guidelines set forth in the Declaration of Helsinki. All participants provided written informed consent to participate. The study design has been described elsewhere (Popov et al. 2017). Briefly, 10 untrained males (median age, 22 years [interquartile range, 21–26 years]; weight, 74 kg [72–79 kg]; and body mass index, 23 kg/m^2^ [22–25 kg/m^2^]) performed a one‐legged continuous knee extension exercise at the same relative intensity before and after 2 months of aerobic training. The intensity of the continuous one‐legged knee extension exercise was chosen by conducting an incremental one‐legged ramp test, which was performed 48 h before each one‐legged continuous knee extension exercise. Biopsy samples were taken from both legs (biological replicates) at rest before and after the 2‐month training period (baseline samples). Additionally, biopsy samples were taken from the exercised leg 1 and 4 h after the one‐legged continuous knee extension exercise (Fig. 1).

All individuals participated in the 2‐month progressive aerobic training program (five sessions/week), which was performed using electromagnetic cycle ergometers (Ergoselect 200, Ergoline, Germany). Before and every second week during the training period, a submaximal incremental two‐legged (cycling) test was performed to evaluate power at a blood lactate concentration of 4 mmol/L (LT_4_) and to correct the training load. The training program comprised training sessions that alternated between continuous (60 min, 70% LT_4_) and intermittent ([3 min, 50% LT_4_ + 2 min, 85% LT_4_] × 12) exercise.

### One‐legged knee extension ramp test

Each participant performed an incremental one‐legged ramp test on a modified electromagnetic ergometer (Ergometric 900S, Ergoline) until exhaustion. The anaerobic threshold (AT) of the *m. vastus lateralis* (measured by electromyography and changes in deoxyhemoglobin content) were evaluated as described previously (Popov et al. 2017). The initial load, load increment, and knee extension rate were 0 W, 2.5 W/min, and 60 cycles/min, respectively. The incremental one‐legged ramp test to exhaustion was repeated 48 h after the final training session of the 2‐month aerobic cycle training program (Fig. 1).

### One‐legged continuous knee extension exercise

Participants were instructed to refrain from exercise for a period of 48 h after the incremental one‐legged ramp test. After that, they arrived at the laboratory at 07:00 and ate a standardized breakfast (3582 kJ; 22 g protein, 154 g carbohydrate, and 16 g lipids). At 09:15 (after a 30‐min rest in the supine position), a baseline sample was taken from the *m. vastus lateralis* of each leg. At 09:35, participants began the one‐legged continuous knee extension exercise (5‐min warm up [60% of AT], followed by 55 min at 75% AT). Participants ate a standardized lunch (3714 kJ; 45 g protein, 183 g carbohydrate, and 27 g lipids) 1 h 20 min postexercise. Biopsies were taken from the *m. vastus lateralis* of the exercised leg prior to and at 1 and 4 h postexercise.

Muscle samples were taken under local anesthesia (2 mL 2% lidocaine) using a microbiopsy technique (Hayot et al. 2005). The samples were blotted quickly with gauze to remove superficial blood, frozen in liquid nitrogen, and stored at −80°C until required. The one‐legged continuous knee extension exercise was repeated 48 h after the second incremental one‐legged ramp test (after the 2‐month cycling training program; Fig. 1).

### Measurement of mitochondrial respiration

Mitochondrial respiration was measured at rest prior to exercise before and after the 2‐month cycling training program as described elsewhere (Popov et al. 2017). A piece of each fresh biopsy sample was placed immediately in ice‐cold relaxing buffer (BIOPS), and the fiber bundles were separated using a pair of needles. Then, the fibers were incubated for 30 min in 2 mL BIOPS buffer containing saponin (50 *μ*g/mL). The fibers were washed twice (10 min each time) in ice‐cold MiR05 buffer. The mass‐specific oxygen consumption rate of ~1.5 mg (wet weight) muscle fibers in 2 mL buffer MiR05 was then measured at 37°C using an Oxygraph Plus System (Hansatech, UK). To avoid potential oxygen limitation, all experiments were carried out under hyperoxygenated conditions (CO_2_ > 180 *μ*mol/L). State 2 respiration (absence of adenylates) was assessed by adding malate (2 mmol/L), pyruvate (5 mmol/L), and glutamate (10 mmol/L). Maximal coupled respiration with convergent electron input to complexes I and II of the electron transport system (State 3) was achieved by adding ADP (5 mmol/L) in MgCl_2_ (0.6 per 1 mol/L ADP), followed by addition of succinate (10 mmol/L). The integrity of the outer mitochondrial membrane was tested by adding cytochrome c (10 *μ*mol/L). If respiration remained stable, the quality of the mitochondria was considered sufficient. Next, oligomycin (2 *μ*g/mL) was added to block complex V (leak state). Finally, an uncoupler (trifluoromethoxy carbonylcyanide phenylhydrazone; FCCP) was added at increasing concentrations (from 0.25 to 1.25 *μ*mol/L).

### Western blot analysis

Western blot analysis is described elsewhere (Popov et al. 2017). Briefly, frozen tissue samples (~10 mg) were homogenized in ice‐cold RIPA buffer containing phosphatase and protease inhibitors (50 mmol/L *β*‐glycerophosphate, 50 mmol/L NaF, 1 mmol/L Na_3_VO_4_, 20 mg/L aprotinin, 50 mg/L leupeptin, 20 mg/L pepstatin, and 1 mmol/L PMSF). The samples were then centrifuged for 10 min (16,000*g*) at 4°C, and the protein concentration in the supernatants was measured in a bicinchoninic acid assay. Next, samples were mixed with Laemmli buffer and loaded onto 7.5–10% polyacrylamide gels (20 *μ*g protein/lane). Electrophoresis (20 mA per gel) was performed using a Mini‐PROTEAN Tetra Cell system (Bio‐Rad, USA). The proteins were transferred to nitrocellulose membranes using a Trans‐Blot Turbo system (Bio‐Rad) in Towbin buffer for 30 min at 25 V. The membranes were stained with Ponceau S to verify consistent loading of proteins, followed by washing and incubation for 1 h in 5% nonfat dry milk. Next, the membranes were incubated at 4°C overnight with antibodies specific for phospho‐ACC1/2^Ser79/222^ (1:1000; ab68191; Abcam, UK), phospho‐p38 MAPK^Thr180/Tyr182^ (1:500; ab195049; Abcam), phospho‐CaMKII^Thr286^ (1:2500; ab32678; Abcam), phospho‐CREB1^Ser133^ (1:500; ab32096; Abcam), phospho‐FOXO1^Ser256^ (1:1000; sc‐101681; Santa Cruz Biotechnology, USA), ESRRG (1:500; ab128930; Abcam), PGC‐1*α* (1:500; ST2102; Calbiochem, and 1:500; ab54481; Abcam [the same results were obtained; therefore, only data obtained using the Calbiochem antibody were reported]), NR4A3 (1:1000; ab41918; Abcam), TFAM (1:1000; ab119684; Abcam), CRTC2 (1:500; 13017; Cell Signaling Technology), NCOR1 (1:500; ab24552; Abcam), and oxidative phosphorylation proteins (OXPHOS) NDUFB8, SDHB, UQCRC2, COX1, and ATP5A1 (1:2500; ab110413; Abcam). Blots were then incubated for 1 h at room temperature with an anti‐rabbit Ig secondary antibody (Cell Signaling Technology). After each step, the membranes were washed three times for 5 min with PBS–Tween 20. Finally, the membranes were incubated with ECL substrate (Bio‐Rad) or with SuperSignal West Femto Maximum Sensitivity Substrate (Thermo Scientific, USA) and luminescent signals were captured using a ChemiDoc Imaging System (Bio‐Rad). All data are expressed as the ratio of target protein to the loading control (as evaluated by Coomassie blue staining).

### Expression of myosin heavy chain expression

Frozen tissue samples were homogenized in 10 volumes of 4% SDS and centrifuged for 10 min at 16,000*g*. The supernatant was mixed with sample buffer (5% *β*‐mercaptoethanol, 2.5% SDS, 10% glycerol, 62.5 mmol/L Tris [pH 6.8], and 0.1% bromophenol blue), boiled for 2 min and separated in acrylamide gels (separating gel: 30% glycerol, 8% acrylamide, 200 mmol/L Tris‐HCl [pH 8.8], 100 mmol/L glycine, and 0.4% SDS; stacking gel: 30% glycerol, 4% acrylamide, 70 mmol/L Tris‐HCl [pH 6.80], 4 mmol/L EDTA, and 0.4% SDS) using a Mini‐PROTEAN Tetra Cell. Gels were stained with Coomassie blue, and myosin heavy chain isoform expression (MHCI and MHCIIA + MHCIIX) was visualized using a ChemiDoc Imaging System.

### Real‐time PCR

RNA extraction and PCR protocols are described elsewhere (Popov et al. 2017). Briefly, RNA was extracted from frozen samples (~20 mg) using an RNeasy mini kit (Qiagen). After DNase (Fermentas) treatment, an MMLV RT kit (Evrogen) was used to obtain cDNAs using a mix of random and oligo (dT) primers and 1 *μ*g total RNA. Real‐time PCR was performed using a Rotor‐Gene Q cycler (Qiagen) using qPCRmix‐HS SYBR (Evrogen). The specificity of the amplification was monitored by analysis of melting curves and gel electrophoresis. Expression of mRNA‐encoding target genes was calculated using the efficiency‐corrected ∆Ct method (Livak and Schmittgen 2001) as ${\left(1+E_{\mathrm{ref}}\right)}^{ct_{\mathrm{ref}}}/{\left(1+E_{\mathrm{tar}}\right)}^{ct_{\mathrm{tar}}}$. PCR efficiency (*E*) was calculated using standard curves for target and reference genes. Calculations revealed that the 2‐month training program altered baseline expression of typical reference genes *GAPDH* and *RPLP0* (*P* < 0.05). Eisenberg and Levanon examined RNA‐sequencing data to determine expression of many potential reference genes in several human tissues (Eisenberg and Levanon 2013); based on the results of that study, *CHMP2A* was chosen as a reference gene in the present study. Here, expression of *CHMP2A* mRNA did not change after training or after acute exercise. The primer sequences used for PCR are shown in Table S1.

In addition, mRNA responses to acute exercise were compared with those reported by other studies investigating transcriptome responses to acute aerobic exercise in human *m. vastus lateralis* (five microarray studies and one RNA‐sequencing study; see Table S2).

### Statistical analysis

Because the sample size was small (*n* = 10) and the data were not normally distributed, all data are expressed as the median and interquartile range. The Wilcoxon matched‐pairs signed‐rank test was used to compare measurements, and Holm–Bonferroni correction was applied to repeated measurements. A *P*‐value of <0.05 was deemed significant.

## Results

### Physiological effects of the 2‐month training program

Training on a cycling ergometer increased aerobic performance in the two‐ and one‐legged incremental tests: the AT increased by 35% (*P* < 0.01) and 17% (*P* < 0.01), respectively. Muscle tissue (samples taken at rest prior to exercise) showed an increasing trend (~1.5‐fold) in terms of baseline ADP‐stimulated mitochondrial respiration in permeabilized fibers (*n* = 6; *P* = 0.06; Table 1).

**Table 1:** Effect of a 2‐month aerobic training program on physiological parameters

	UT	ET	*P*‐value
Incremental ramp test (*n* = 10)			
Lactate threshold in two‐legged test, W	145 (118–185)	195 (175–246)	<0.01
Anaerobic threshold in one‐legged test, W	38 (34–44)	45 (42–53)	<0.01
Respiration in permeabilized muscle fibers (*n* = 6)			
Malate + Pyruvate + Glutamate, pmol/sec/mg	8.3 (6.8–13.5)	2.7 (1.7–6.9)	NS
ADP, pmol/sec/mg	15 (13–19)	26 (20–39)	0.06
Succinate, pmol/sec/mg	31 (29–43)	43 (33–57)	0.06
Cytochrome c, pmol/sec/mg	28 (24–44)	36 (30–55)	NS
Oligomycin, pmol/sec/mg	29 (25–34)	24 (15–28)	NS
FCCP, pmol/sec/mg	35 (31–41)	56 (37–70)	NS

Data are expressed as the median (interquartile range).

UT, untrained; ET, endurance trained; NS, nonsignificant.

### Baseline (at rest prior to exercise) phosphorylation

**Figure 2:**
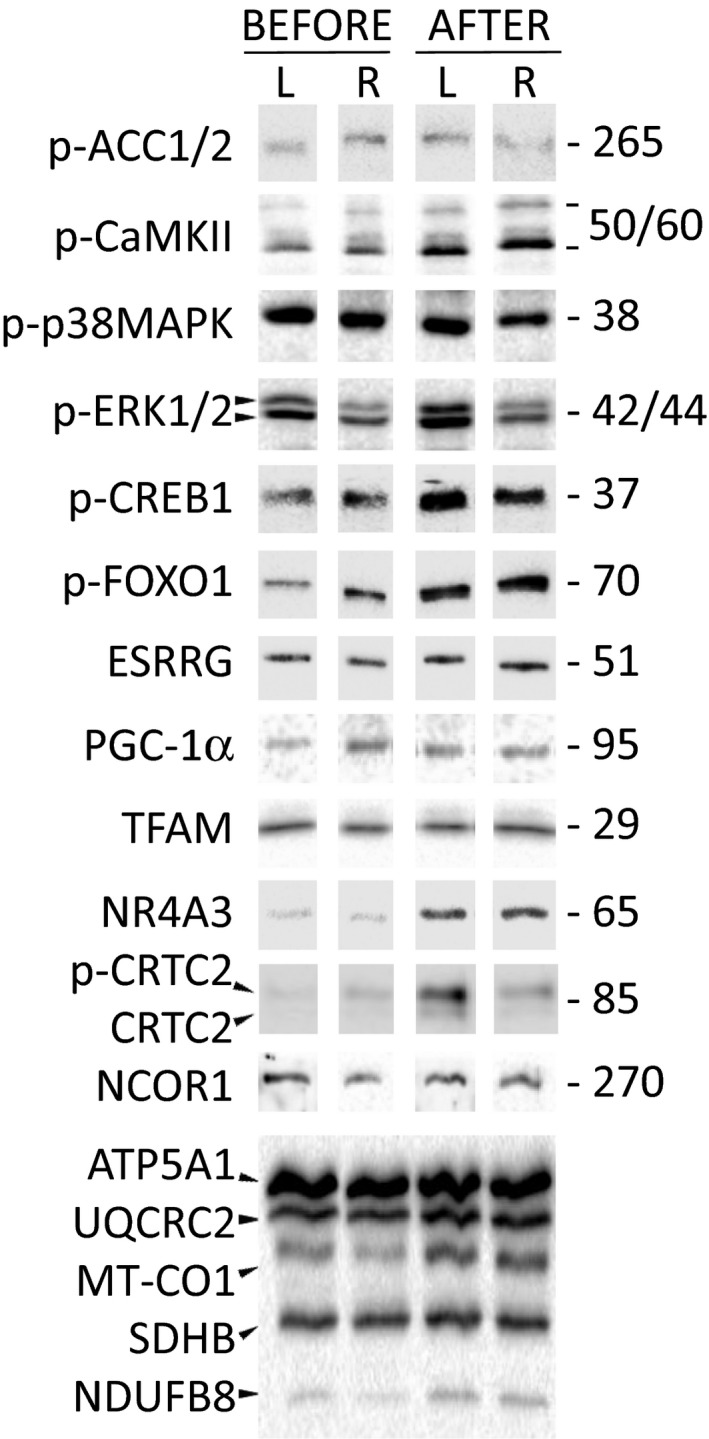
Representative immunoblots. Level of proteins in left (*L*) and right (*R*) legs (biological replicates) at rest *before* and *after* the 2‐month training program.

**Figure 3:**
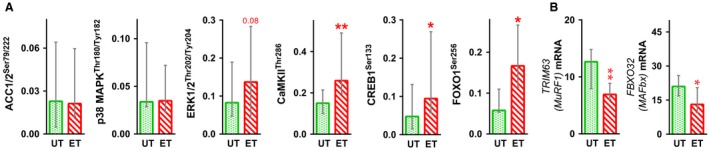
Effect of a 2‐month aerobic training program on baseline phosphorylation and gene expression. Baseline phosphorylation of kinases ACC1/2^Ser79/222^ (a marker of AMPK activity), CaMKII^Thr286^, p38 MAPK^Thr180/Tyr182^ and ERK1/2^Thr202/Tyr204^, and transcription factors CREB1^Ser133^ and FOXO1^Ser256^ (A). Baseline expression of FOXO1‐related genes *TRIM63* (*MURF1*) and *FBXO32* (*MAFbx*) (B). UT, untrained; ET, endurance trained. Data represent the median (interquartile range). *P < 0.05 and **P < 0.01, compared with untrained state; *n* = 9–10.

Training increased baseline phosphorylation of CaMKII^Thr286^ (by 1.7‐fold, *P* < 0.01), CREB1^Ser133^ (by twofold, *P* < 0.05) and FOXO1^Ser256^ (by 2.9‐fold, *P* < 0.05); phosphorylation of ERK1/2^Thr202/Tyr204^ tended to increase (by 1.7‐fold, *P* = 0.08). Baseline phosphorylation of ACC1/2^Ser79/222^ (a marker of AMPK activity) and p38 MAPK^Thr180/Tyr182^ did not change (Figs. 2 and 3A).

### Baseline (at rest prior to exercise) protein levels

**Figure 4:**
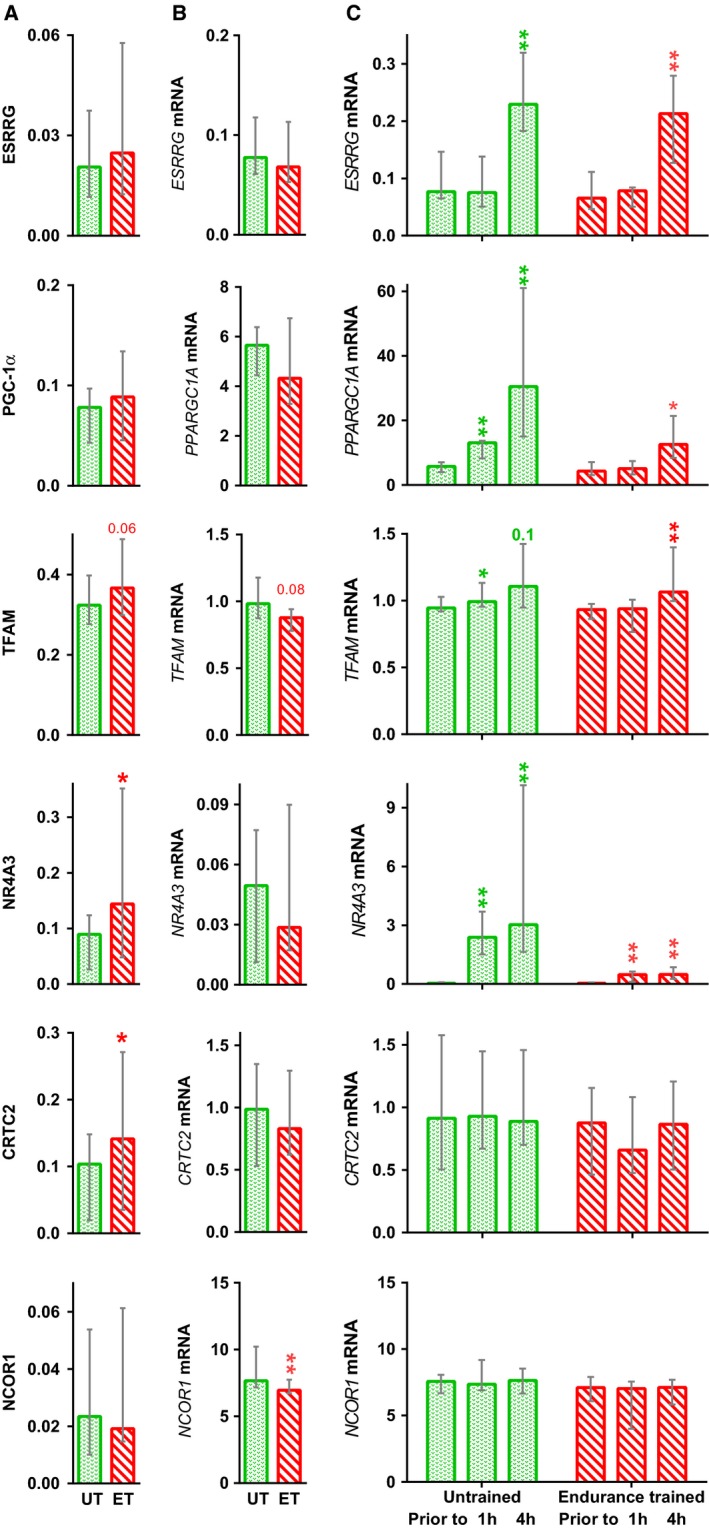
Effect of a 2‐month aerobic training program on expression of transcriptional regulators. Baseline level of proteins (A) and mRNA (B), and mRNA responses to acute exercise (C), in untrained (UT) and endurance trained (ET) muscle. Data represent the median (interquartile range). *P < 0.05 and **P < 0.01, compared with untrained state; *n* = 9–10.

**Figure 5:**
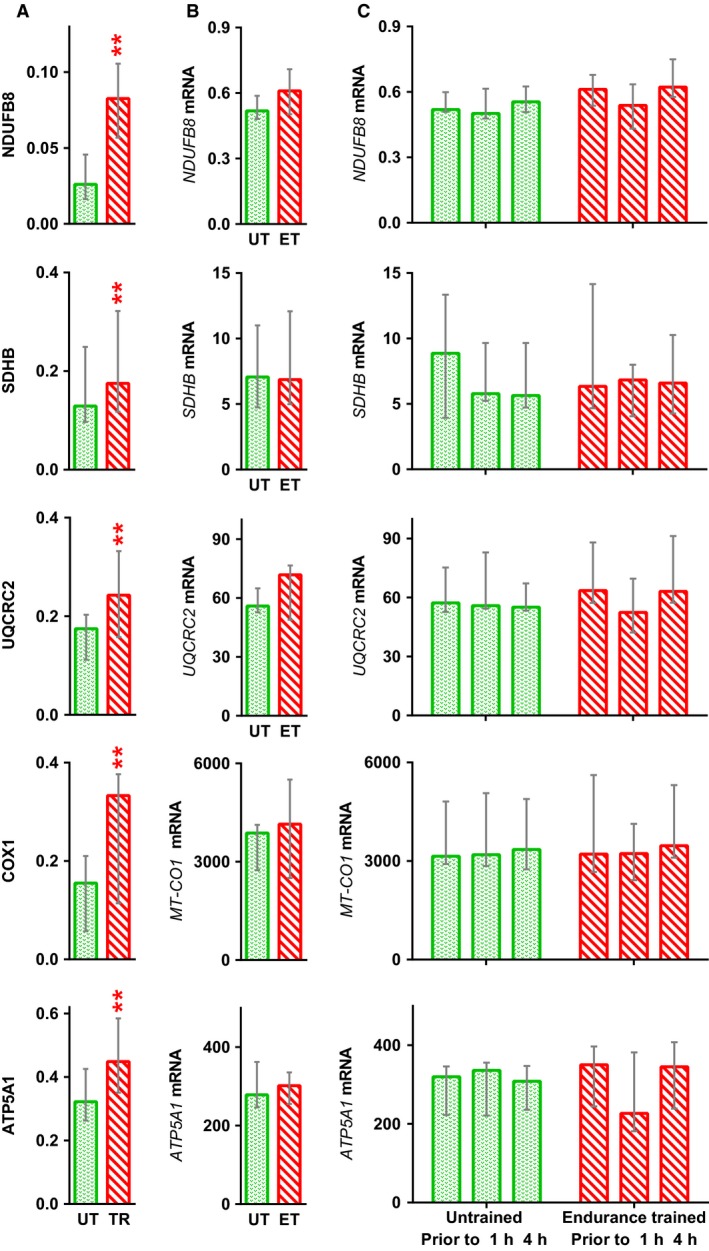
Effect of a 2‐month aerobic training program on expression of mitochondrial respiratory complex proteins. Baseline level of proteins (A) and mRNA (B), and mRNA responses to acute exercise (C), in untrained (UT) and endurance trained (ET) muscle. Data represent the median (interquartile range). **P < 0.01, compared with untrained; *n* = 9–10.

Prior to training, the relative amount of myosin heavy chains types I and II (IIA + IIX) in muscle was 32 (29–41)% and 71 (60–77)%, respectively. Two months of aerobic training did not affect expression of myosin heavy chains. Training increased the amounts of transcriptional regulators NR4A3 (by 1.4‐fold, *P* < 0.05) and CRTC2 (by 1.4‐fold, *P* < 0.05). In addition, TFAM content tended to increase (by 1.2‐fold, *P* = 0.06). Baseline levels of PGC‐1*α*, ESRRG, and NCOR1 did not change (Fig. 4A). Training increased (by ~1.4–3‐fold, *P* < 0.01; Fig. 5A) baseline levels of mitochondrial proteins (NDUFB8, SDHB, UQCRC2, COX1, and ATP5A1) in respiratory complexes I–V.

### Gene expression under baseline conditions (at rest prior to exercise) and after exercise

After 2 months of training, baseline expression of FOXO1‐dependent E3 ligases *TRIM63* (*MURF1*) and *FBXO32* (*MAFbx*) fell (by twofold [*P* < 0.01] and 1.7‐fold [*P* < 0.05], respectively; Fig. 3B).

Baseline expression of *NCOR1* mRNA fell by 10% (*P* < 0.01). Baseline expression of other genes encoding transcription regulators and proteins within respiratory complexes did not change (Fig. 4B).

Acute aerobic exercise increased expression of genes encoding regulators of mitochondrial biogenesis (*NR4A3* [*P* < 0.01], *ESRRG* [*P* < 0.01], *PPARGC1A* [all *P* < 0.01–0.05], and *TFAM* [*P* < 0.05]) in both untrained and trained muscle, but did not change expression of *CRTC2* and *NCOR1* mRNA (Fig. 4B and C). Expression of genes encoding proteins within respiratory complexes I–V (*NDUFB8*,* SDHB*,* UQCRC2*,* MT‐CO1,* and *ATP5A1*) did not change (Fig. 5B and C).

## Discussion

Two months of aerobic training increased the aerobic capacity of the knee‐extensor muscles, the maximum rate of ADP‐stimulated mitochondrial respiration and amounts of respiratory proteins within complexes I–V. The amounts of some signaling proteins and expression levels of some genes in type I muscle fibers may differ from those in type II muscle fibers (van Wessel et al. 2010). Here, we found that expression of myosin heavy chains types I and II did not change after training; therefore, training‐induced changes in the protein content and expression of related genes were not associated with changes in myosin phenotype.

### Basal phosphorylation

We found no differences in baseline phosphorylation of ACC1/2^Ser79/222^ (a marker of AMPK activity) between untrained and trained muscle; this agrees with other data regarding baseline activity of AMPK *α*2 and phosphorylation of ACC1/2^Ser79/222^ in untrained and trained human muscle (Nielsen et al. 2003; Yu et al. 2003; Lee‐Young et al. 2009), but contradicts the data of one study showing an increase in baseline ACC1/2^Ser79/222^ phosphorylation and AMPK *α*2 activity in trained muscle (Frosig et al. 2004). After training, the baseline level of p38 MAPK^Thr180/Tyr182^ phosphorylation remained unchanged, whereas that of ERK1/2^Thr202/Tyr204^ tended to increase relative to that in untrained muscle. This is, in part, consistent with the results of a previous study reporting no difference in p38 MAPK^Thr180/Tyr182^ and ERK1/2^Thr202/Tyr204^ phosphorylation in the muscles of trained and untrained males (Yu et al. 2003).

Aerobic training increased baseline phosphorylation of some kinases and transcription factors associated with muscle adaptation to contractile activity. Baseline phosphorylation of CaMKII^Thr286^ and CREB1^Ser133^ in trained muscle was higher than that in untreated muscle, a finding consistent with those of previous studies of volunteers (Rose et al. 2007) and rodents (Feng et al. 2013). Some studies suggest that adaptations in baseline activation of CaMKII and phosphatase calcineurin in muscles subjected to prolonged low‐intensity contractile activity may be associated with an increase in the baseline content of free Ca^2+^ in the cytoplasm (Bruton et al. 2010; Tavi and Westerblad 2011; Zhang et al. 2014). Ca^2+^‐dependent signaling (Handschin et al. 2003; Long et al. 2007; Roberts‐Wilson et al. 2010), in particular an increase in the baseline content of free Ca^2+^ in the cytoplasm (Wright et al. 2007; Bruton et al. 2010), plays a role in regulating mitochondrial biogenesis in myoblasts and rodent muscle. Ca^2+^‐dependent regulation of mitochondrial biogenesis is (partially) associated with expression of transcription factors belonging to the ATF/CREB family, with an increased expression of *PPARGC1A* (Handschin et al. 2003; Wright et al. 2007) and *NR4A3* (Goode et al. 2016). ATF/CREB family proteins form heterodimers with a number of proteins from the AP1 family, and with some proteins containing the bZIP domain (Hai and Curran 1991; Newman and Keating 2003); these may be involved in regulating many genes. Therefore, an increase in the baseline phosphorylation of CREB1^Ser133^ in trained muscle may be associated with both an increase in baseline gene expression and with the presetting of this transcriptional regulator to respond to subsequent acute stress (e.g., acute exercise).

FOXO family proteins play an important role in regulating metabolism and in ubiquitin‐ and autophagy–lysosomal‐dependent proteolysis within skeletal muscle (Sanchez et al. 2014). In human skeletal muscle, aerobic exercise leads to a transient fall in FOXO1^Ser256^ phosphorylation (an activation marker) and an increase in expression of FOXO1‐dependent genes encoding muscle‐specific E3 ligases (Harber et al. 2009; Luden et al. 2010; Pagano et al. 2014; Popov et al. 2014; Stefanetti et al. 2015; Lysenko et al. 2018). By contrast, we found that aerobic training increased basal FOXO1^Ser256^ phosphorylation (a deactivation marker) and reduced basal expression of the E3 ligases *TRIM63* (*MURF1*) and *FBXO32* (*MAFbx*). An increase in baseline FOXO1^Ser256^ phosphorylation may be associated with an increase in the baseline activity of protein kinase B (AKT); indeed, a previous study showed that baseline AKT^Ser473^ phosphorylation human *m*. *vastus lateralis* after a period of aerobic training is higher than that before training (Wilkinson et al. 2008). It seems that FOXO‐dependent activation of muscle proteolysis in the first hours after exercise is important for removal of damaged proteins, whereas a fall in baseline activity of this pathway can play an important role in reducing the baseline rate of proteolysis and in maintaining a high rate of muscle protein synthesis, a process responsible for adaptation of skeletal muscle to aerobic training.

To summarize, previous studies show that phosphorylation and activity of the kinases and transcription factors described above can change in response to acute exercise. Here, we showed that aerobic training increases baseline phosphorylation of CaMKII^Thr286^ and ERK1/2^Thr202/Tyr204^ as well as that of CREB1^Ser133^ and FOXO1^Ser256^.

### Baseline levels of transcriptional regulators

Transcription factors ESRRA and ESRRG bind to the promoters of many genes encoding mitochondrial proteins (Dufour et al. 2007); their activity is regulated by the coactivator PGC‐1*α* (Hentschke et al. 2002; Baresic et al. 2014). Transcriptomic studies (Neubauer et al. 2013; Vissing and Schjerling 2014; Popov et al. 2018) show that, in contrast to mouse muscle (Rangwala et al. 2010), aerobic exercise induces expression of *ESRRG* in human muscle but does not affect expression of *ESRRA*. Overexpression of *Esrrg* in mouse skeletal muscle increases aerobic performance and $\dot{V}$o_2max_, which is associated with an increase in capillarization, mitochondria volume density, and expression of genes regulating oxidative, carbohydrate, and lipid metabolism via PGC‐1*α*‐dependent and ‐independent mechanisms (Rangwala et al. 2010; Narkar et al. 2011). Here, we show that acute exercise increased expression of *ESRRG* inf both untrained and trained muscle; however, there was no change in baseline expression of *ESRRG* mRNA and protein after 2 months of training. Similar dynamics were observed for *PPARGC1A* mRNA and protein, a well‐characterized coactivator playing an important role in adaptation of skeletal muscles to aerobic exercise (Scarpulla 2008; Olesen et al. 2010). It can be assumed that the transient but significant increase in expression of *ESRRG* and *PPARGC1A* at 4 h postacute exercise increases expression of these proteins at later stages of recovery. In this case, the lack of increase in the basal amounts of these proteins after training can be explained by the high rate of degradation (Puigserver et al. 2001; Sano et al. 2007; Olson et al. 2008; Ren et al. 2011). The lack of increase in the baseline amounts of these proteins reported herein (as opposed to the increases reported by (Lanza et al. 2008; Irving et al. 2015; Egan et al. 2013)) may be explained by differences in the training protocol and/or by a training period (2 months) that was too short.

TFAM is necessary for transcription of mitochondrial DNA and for mitochondrial biogenesis. Knocking out *Tfam* in mouse cardiac muscle results in a lethal outcome during the neonatal period (Li et al. 2000), whereas overexpression of *TFAM* in cardiac myocytes increases both mitochondrial DNA copy number and the amount of respiratory enzymes (Fujino et al. 2012; Ikeda et al. 2015). PGC‐1*α* coactivator and its partners nuclear respiratory factors 1 and 2 play an important role in regulating *TFAM* expression (Scarpulla 2008). More than ten studies show that aerobic exercise increases expression of *TFAM* mRNA in human *m*. *vastus lateralis* (see review (Islam et al. 2018)); aerobic training increases baseline expression of the TFAM protein (Norrbom et al. 2010; Bori et al. 2012; Granata et al. 2016). Here, we show that training increased baseline expression of this protein without increasing basal expression of the *TFAM* gene, a finding consistent with that of previous work (Norrbom et al. 2010). Therefore, the training‐induced increase in baseline expression of TFAM protein is associated with a transient increase in *TFAM* mRNA expression after each exercise session in both untrained and trained muscle.

The functions of transcription factor NR4A3 and mechanisms regulating *NR4A3* gene expression are comparable which those of PGC‐1*α*. In rodent skeletal muscle, NR4A3 regulates carbohydrate and lipid metabolism, oxidative phosphorylation, angiogenesis, and lysosomal proteolysis. Mice overexpressing *Nr4a3* in muscle show increased muscle glycogen stores, mitochondrial volume density, capillarization, running time to exhaustion, and rate of fat oxidation (Pearen et al. 2012, 2013; Goode et al. 2016). In rat muscle, the basal level of the NR4A3 protein does not change after 3 weeks of aerobic training (Kawasaki et al. 2011); however, the basal amount of NR4A3 in muscles of rats selected for their endurance (with respect to running) is higher than that in animals with low endurance (Stephenson et al. 2012). We showed that aerobic training increases the baseline amount of NR4A3 protein in human muscle. This increase is not associated with a change in baseline expression of the *NR4A3* gene; rather, it depends on increased expression of this gene in both untrained and trained muscle after acute exercise.

CRTC coactivators play an important role in adaptation of skeletal muscles to aerobic exercise. CRTCs regulate the transcriptional activity of CREB1 (Altarejos and Montminy 2011); overexpression of the *Crtc* gene in mouse skeletal muscle increases expression of a number of mitochondrial genes and the maximum rate of mitochondrial respiration via the PGC‐1*α*‐dependent mechanism (Wu et al. 2006). We found that 2 months of training increased the baseline amount of CRTC2 in human muscle. This increase was not associated with a change in expression of its gene (either in baseline or after acute exercise) in untrained and trained muscle. Analysis of public transcriptomic data also revealed no change in expression of the *CRTC2* gene in human *m*. *vastus lateralis* at 0.5, 2.5, 3, 4, 5, 8, 48, or 96 h after aerobic exercise (Table S2). Training‐induced increases in baseline expression of CRTC2 were associated with changes in the rate of its synthesis or degradation.

The transcriptional corepressor NCOR1 binds to several transcription factors, including PPARD and ESRRs, and inhibits their activity. Knocking out *Ncor1* in mouse skeletal muscle increases expression of oxidative enzymes; it also increases the numbers of mitochondria and capillaries, the rate of fat oxidation, locomotor activity, and $\dot{V}$o_2max_ (Yamamoto et al. 2011; Pérez‐Schindler et al. 2012). In mouse muscle, expression of the *Ncor1* gene fell at 3‐h postaerobic exercise (Yamamoto et al. 2011); moreover, analysis of the transcriptome in human *m. vastus lateralis* after aerobic exercise predicted a fall in the transcriptional activity of NCOR1 (Popov et al. 2018). However, we found no reduction in expression of *NCOR1* after acute exercise, which is consistent with the result of another study (Gidlund et al. 2015) and with the majority of data from transcriptomic studies (Table S2). We found a significant, but very small (~10%), decrease in baseline expression of *NCOR1* and did not detect any change in baseline expression of NCOR1. It is assumed that the potential effects of NCOR1 in human skeletal muscle are realized through other mechanisms; for example, translocation of NCOR1 from the nucleus to the myoplasm (Yamamoto et al. 2011).

### Baseline levels of mitochondrial proteins in respiratory complexes

Training increased the amounts of mitochondrial proteins (NDUFB8, SDHB, UQCRC2, COX1, and ATP5A1) in respiratory complexes I–V in muscle without altering gene expression at baseline or after acute exercise. Analysis of public transcriptomic data (Table S2) confirmed no increase in expression of *NDUFB8*,* SDHB*,* UQCRC2,* or *ATP5A1* in human *m. vastus lateralis* at 0.5, 2.5, 3, 4, 5, 8, 48, or 96 h after aerobic exercise. Moreover, in HeLa cells, misfolding stress induces an increase in expression of ATP synthase subunits and other mitochondrial respiratory proteins without altering expression of the genes encoding these proteins (Cheng et al. 2016). Therefore, it is assumed that an increase in the amounts of these proteins in respiratory complexes is not regulated at the transcriptional level; rather, it is related to regulation of the rate of synthesis and degradation (see below). Despite the fact that a transient increase in gene expression is expected within a few hours after exercise, we cannot exclude the possibility that this could happen at later stages of recovery. For example, increased expression of some genes encoding mitochondrial enzymes (*HADH*,* ALAS1*,* CPT1*) and electron carriers (*CYCS*) in human *m*. *vastus lateralis* was detected between 10 and 24 h after aerobic exercise (Leick et al. 2010). These data emphasize the relevance of future transcriptomic studies conducted during this recovery period.

Interestingly, we found that training‐induced increases in the content of proteins with varying functions were related to differential regulation at the transcriptional level. There was a transient increase in expression of genes encoding regulators of mitochondrial biogenesis after acute exercise, whereas genes encoding proteins within respiratory complexes demonstrated no increase in expression, either after acute exercise or at baseline. Such differences in regulation may be associated with the different functions of these proteins. This assumption is consistent with the results of a study that used high‐throughput methods to investigate the stability of a variety of mRNAs and their proteins in mouse fibroblast cultures (Schwanhausser et al. 2011). It turns out that mRNA and protein of enzymes that function in the Krebs cycle and respiration are stable, whereas mRNA and protein belonging to transcription regulators are not. These results are in good agreement with our data concerning differences in regulation of respiratory complex proteins and transcription regulators at the transcriptional level.

This study shows that aerobic training alters baseline phosphorylation of kinases and transcription factors (CaMKII^Thr286^, CREB1^Ser133^, and FOXO1^Ser256^) that play an important role in adaptation of skeletal muscle to acute exercise and increases baseline expression of mitochondrial respiratory proteins and some regulators of mitochondrial biogenesis (TFAM, NR4A3, and CRTC2). An increase in the content of these proteins at baseline was not associated with a change in baseline expression of their genes. The increase in the content of regulators of mitochondrial biogenesis (TFAM and NR4A3) at baseline was associated with a transient increase in transcription after acute exercise. Contrariwise, the increase in the content of respiratory proteins at baseline does not seem to be regulated at the transcriptional level; rather, it is associated with other mechanisms. Taken together, these results suggest that adaptation of skeletal muscle to regular aerobic exercise is associated not only with transient molecular responses to exercise, but also with changes in baseline phosphorylation and expression of regulatory proteins.

## Conflict of Interest

None declared.

## Supporting information




**Table S1.** Primers used in this study.

**Table S2.** Analysis of transcriptomic data from studies investigating response to acute aerobic exercise in human *m*. *vastus lateralis*. The table shows significant log_2_ *FoldChange*; NS – nonsignificant change.
