# Supplementary material for: Effect of aerobic training on baseline expression of signaling and respiratory proteins in human skeletal muscle
Source: Physiol Rep. 2018 Sep 10;6(17):e13868. doi: 10.14814/phy2.13868 (PMC6129775; doi:10.14814/phy2.13868)
Supplement: Supplementary file 1 — Table S1. Primers used in this study. [file PHY2-6-e13868-s001.doc]

**Supplementary file 1.** Primers used in this study.

| Transcript | Strand | Sequence, 5’–3’ | Product size, bp |
| --- | --- | --- | --- |
| *ESRRG* | Forward  Reverse | AGCCTGCAAGGCATTCTTCA  GCCTGGCAGGATTTACGTCT | 108 |
| *PPARGC1A* | Forward  Reverse | CAGCCTCTTTGCCCAGATCTT  TCACTGCACCACTTGAGTCCAC | 101 |
| *NR4A3* | Forward  Reverse | CACTACGGCGTGCGAACCT  CATCGGTTTCGACGTCTCTTGT | 125 |
| *TFAM* | Forward  Reverse | AGATTCCAAGAAGCTAAGGGTGATT  TTTCAGAGTCAGACAGATTTTTCCA | 85 |
| *CRTC2* | Forward  Reverse | CCAACAATGTCACCCACCTT  CGCTGGTCAGTGGACAGTTTA | 77 |
| *NCOR1* | Forward  Reverse | TGGGCTTATGGAGGACCCTA  CTGCCTCTGCGTTTCCCATA | 240 |
| *NDUFB8* | Forward  Reverse | AGAAGGGAAACGTGAAGAAGGT  GTTCCGGGATGCCCTTTGC | 86 |
| *SDHB* | Forward  Reverse | GGAAGGCAAGCAGCAGTAT  AGAACTGCAGGCCCCAGA | 144 |
| *UQCRC2* | Forward  Reverse | CCACAGCTGCTGGAGATGTTA  TGTTCTTGGCAGCTTGGACA | 99 |
| *MT-CO1* | Forward  Reverse | GTCACAGCCCATGCATTTGT  AGTTGCCAAAGCCTCCGATT | 70 |
| *ATP5A1* | Forward  Reverse | TTGCCCAGTTCGGTTCTGAC  GATATCCCCTTACACCCGCA | 150 |
| *TRIM63*(*MURF1*) | Forward  Reverse | CTCAGTGTCCATGTCTGGAGGCCGTT  GGCCGACTGGAGCACTCCTGTTTGTA | 147 |
| *FBXO32*(*MAFbx*) | Forward  Reverse | GTCCAAAGAGTCGGCAAGTC  AGGCAGGTCAGTGAAGGTG | 147 |
| *CHMP2A* | Forward  Reverse | CTGGGACCCTGTCGTCAACAT  AGTAGCTCCTCTGGCGTCTT | 93 |
| *GAPDH* | Forward  Reverse | CAAGGTCATCCATGACAACTTTG  GTCCACCACCCTGTTGCTGTAG | 496 |
| *RPLP0* | Forward  Reverse | CACTGAGATCAGGGACATGTTG  CTTCACATGGGGCAATGG | 77 |
