# Supplementary material for: Effect of aerobic training on baseline expression of signaling and respiratory proteins in human skeletal muscle
Source: Physiol Rep. 2018 Sep 10;6(17):e13868. doi: 10.14814/phy2.13868 (PMC6129775; doi:10.14814/phy2.13868)
Supplement: Supplementary file 2 — Table S2. Analysis of transcriptomic data from studies investigating response to acute aerobic exercise in human m. vastus lateralis. The table shows significant log2 FoldChange; NS – nonsignificant change. [file PHY2-6-e13868-s002.doc]

**Supplementary file 2.** Analysis of transcriptomic data from studies investigating response to acute aerobic exercise in human *m. vastus lateralis*. The table shows significant log_2_ *FoldChange*; NS – non significant change.

| GEO ID | Gene | Time after aerobic exercise | | | | | | | |
| --- | --- | --- | --- | --- | --- | --- | --- | --- | --- |
|  |  | 0.5 h | 2.5 h | 3 h | 4 h | 5 h | 8 h | 48 h | 96 h |
| GSE59088  (microarray) | *NDUFB8 SDHB UQCRC2 MT-CO1 ATP5A1 CRTC2 NCOR1* |  | NS  NS  NS  -  NS  NS  -0.26 |  |  | NS  NS  NS  -  NS  NS  -0.23 |  |  |  |
| GSE43856  (microarray) | *NDUFB8 SDHB UQCRC2 MT-CO1 ATP5A1 CRTC2 NCOR1* |  |  | NS  NS  NS  -  NS  NS  NS |  |  |  | NS  NS  NS  -  NS  NS  NS | NS  NS  NS  -  NS  NS  NS |
| GSE43219  (microarray) | *NDUFB8 SDHB UQCRC2 MT-CO1 ATP5A1 CRTC2 NCOR1* | NS  NS  NS  -  NS  NS  NS |  |  |  |  |  |  |  |
| GSE41769  (microarray) | *NDUFB8 SDHB UQCRC2 MT-CO1 ATP5A1 CRTC2 NCOR1* | NS  NS  NS  -  NS  NS  NS |  |  |  |  |  |  |  |
| GSE27285  (microarray) | *NDUFB8 SDHB UQCRC2 MT-CO1 ATP5A1 CRTC2 NCOR1* |  |  | NS  NS  NS  -  NS  NS  NS |  |  |  | NS  NS  NS  -  NS  NS  NS |  |
| GSE86931  (RNA-seq) | *NDUFB8 SDHB UQCRC2 MT-CO1 ATP5A1 CRTC2 NCOR1* |  |  |  | NS  NS  NS  -  NS  NS  NS |  | NS  NS  NS  -  NS  NS  NS |  |  |
